# Alternate Choice Organ Counselling in Altruistic Non‐Directed Solid Organ Donation: An Ethical Analysis

**DOI:** 10.1111/jep.70055

**Published:** 2025-03-25

**Authors:** Richard C. Armitage

**Affiliations:** ^1^ School of Medicine, Academic Unit of Population and Lifespan Sciences, Clinical Sciences Building, Nottingham City Hospital Campus University of Nottingham Nottingham UK

**Keywords:** ethics, health policy, kidney donation, liver donation, organ donation

## Abstract

**Introduction:**

Currently, recruitment of non‐directed altruistic (NDA) kidney and liver lobe donors in the UK regards these individuals as potential NDA donors of the particular organ type they initially express an interest in donating. Conceptualising these individuals instead as potential NDA donors of either a kidney or a liver lobe would require them to be counselled on both kidney donation and liver lobe donation. This can be referred to as ‘alternate choice organ counselling’.

**Methods:**

This paper conducts an ethical analysis of alternate choice organ counselling using the ethical framework of Principlism, and suggests changes to current policy and practice, accordingly.

**Findings:**

This paper finds multiple strong ethical reasons to carry out alternate choice organ counselling for potential NDA donors of kidneys or liver lobes: the duty to respect autonomy requires alternate choice organ counselling such that the potential donor's decision to become a NDA donor of a particular organ type is fully informed; the duty of non‐maleficence requires alternate choice organ counselling such that the harm subjected to the donor through living donation can be minimised (although such counselling might generate serial NDA donors, which would expose them to greater total harm); the asymmetry in the degree to which the living kidney and living liver lobe donation mechanisms promote justice requires alternate choice organ counselling for potential donors who wish to maximise the utility of their single NDA donation; finally, alternate choice organ counselling is likely to promote beneficence in potential NDA donors.

**Discussion:**

This paper finds ethical reasons for potential NDA donors to be conceptualised as potential NDA donors of either a kidney or liver lobe, and for these individuals to be provided with alternate choice organ counselling. Suggestions on how this might be delivered in practice are offered, and the necessary further quantitative and qualitative research outlined.

## 1 Introduction

In the UK, it is possible for living individuals to donate a solid organ—either a kidney or a liver lobe—through the National Health Service (NHS) [1] in a process known as living organ donation. Between 01 April 2023 and 31 March 2024, there were 938 living solid organ donors in the UK [2]. 907 (96.7%) of these individuals donated a kidney, and 31 (3.3%) donated a liver lobe.

Solid organ donation is termed ‘altruistic’ if two conditions are satisfied: first, the donor has no prior relationship with the recipient; second, the donor has not agreed to donate an organ for a loved one to receive an organ from another donor in return [3].^1^ Altruistic donation is either directed or non‐directed [4]: in directed altruistic donation, the organ is donated to a specific individual with whom the donor has no prior relationship (such as the subject of a media campaign); in non‐directed altruistic (NDA) donation, the organ is donated to an unspecified individual with whom the donor has no prior relationship.

In the UK, both living NDA kidney donation and NDA liver lobe donation are available. Between 01 April 2023 and 31 March 2024, there were 48 NDA kidney donations in the UK [5], meaning 5.3% of the living kidney donations during this period were of this kind. The number of NDA liver lobe donations during the same period is not publicly available, but must be substantially lower than the number of NDA kidney donations since the total number of liver lobe donations of all kinds was 31.

## 2 Recruitment of NDA Solid Organ Donors in the UK

Currently, NDA solid organ donors in the UK are recruited via distinct pathways that are specific to the type of organ that is to be donated—individuals who are interested in becoming NDA kidney donors make contact with their regional Kidney Donor Team [4], while individuals who are interested in becoming NDA liver lobe donors make contact with their regional Liver Donor Team [6]. Potential donors are then counselled on becoming a NDA donor of the particular organ type they have expressed an interest in donating, and are then subjected to a rigorous process of assessment to evaluate their suitability to become a NDA donor of that particular organ type—specifically, the Kidney Donor Team assesses the suitability of potential NDA kidney donors, while the Liver Donor Team assesses the suitability of potential NDA liver lobe donors. While this process for each organ type involves thoroughly counselling the individual about becoming a NDA donor of that particular organ type, at no point in either process is the potential donor counselled on the possibility of becoming a NDA donor of the other organ type instead i.e. potential NDA kidney donors are only counselled about becoming NDA kidney donors and not about becoming NDA liver lobe donors instead, and potential NDA liver lobe donors are only counselled about becoming NDA liver lobe donors and not about becoming NDA kidney donors instead [7, 8].

Both directed altruistic donors and NDA donors (of kidneys and liver lobes) have no prior relationship with the recipient, and have not agreed to donate in order for a loved one to receive an organ in return. Because directed altruistic donors donate to a specific individual who requires a particular type of organ (either a kidney or liver lobe, as determined by the particular medical condition from which they are suffering), the type of organ to be donated in this form of donation is pre‐determined and is not subject to change. However, the potential exists for NDA donation to be somewhat different because, in this form of donation, donors donate to an unspecified individual. As both NDA donation of both kidneys and liver lobes is available, and because the overall group of unspecified recipients contains both those in need of a donor kidney and those in need of a donor liver lobe, the type of organ to be donated by NDA donors must not necessarily be predetermined and might in fact be subject to change.

Currently, because NDA donors are recruited via distinct pathways that are specific to the type of organ that is to be donated, and because these donors are not counselled on the possibility of becoming a NDA donor of the other organ type instead, the organ type which is donated by a NDA donor is artificially restricted to the particular organ type that is dealt with by the Donor Team they originally approached. While the donor might have approached their regional Kidney Donor Team or Liver Donor Team while both being fully aware of, and having being fully counselled about, the possibility of becoming a NDA donor of the other organ type, it is possible that such donors are entirely unaware of the possibility of donating the other organ type, or have only been partially counselled on this process beforehand.

Accordingly, recruitment of NDA donors currently regards these individuals as potential NDA donors of the particular organ type they initially express an interest in donating. However, it is possible that these individuals could instead be conceptualised as potential NDA donors of either a kidney or a liver lobe. Regarding potential donors in this way would require them to be thoroughly counselled on the processes of both living kidney donation and living liver lobe donation, such that they can subsequently choose which (if any) of these organs to donate as NDA donors. This can be referred to as ‘alternate choice organ counselling.’

This paper will conduct an ethical analysis of alternate choice organ counselling using the ethical framework of Principlism, and suggest changes to current policy and practice, accordingly.

## 3 Principlism

Principlism is the normative ethical framework of professional ethics that was devised by Beauchamp and Childress in 1979 [9]. This framework, which was designed to aid ethical decision‐making in healthcare contexts, comprises of four basic and universal ethical principles that state prima facie moral obligations that are equally important for doctors in the provision of patient care. The Four Principles are the duties of respect for autonomy, beneficence, justice, and non‐maleficence. Since their introduction, the Four Principles have become the primary method for the teaching and evaluation of medical ethical dilemmas in healthcare contexts and [10], due to their strong support [11, 12, 13, 14], are still widely used today.

## 4 Ethical Analysis of Alternate Choice Organ Counselling

### 4.1 Autonomy

Autonomy is the principle that individuals have the right to make decisions, hold views, and undertake actions based on their personal views and values. Broadly speaking, a person is autonomous if they govern their own decisions and actions. Accordingly, autonomy requires the doctor to respect the patient's capacity for self‐determination, and their capacity to make independent decisions about their life in the absence of undue pressure, solicitation or coercion. Fundamentally, a failure to respect a patient's autonomy involves interfering with their capacity to make autonomous choices, or interference with the patient's opportunity to act upon those choices, or both.

Respect for autonomy generates in doctors both ‘negative duties’—which requires them to avoid performing acts that would interfere with the patient's autonomy such as influencing the patient through manipulation, deception, coercion, threats or undue incentives—and ‘positive duties’—which requires them to perform certain acts such as providing opportunities for patients to make their own choices, enabling patients to act on their own choices, and providing patients with understandable information regarding their health and medical treatment. For patients to make truly autonomous choices their consent to make those choices must be valid, which requires them to have access to and understand the relevant information pertaining to each choice.

The current manner in which NDA donors are recruited, in which these individuals are regarded as potential NDA donors of the particular organ type they initially express an interest in donating, generates the positive duty to thoroughly counsel the individual about becoming a NDA donor of that particular organ type (indeed, this is an integral part of current recruitment practices) [7, 8]. However, if these individuals were instead conceptualised as potential NDA donors of either a kidney or a liver lobe, this would generate the broader positive duty of alternate choice organ counselling, in which the individual is thoroughly counselled on the processes of both kidney donation and liver lobe donation. Because potential NDA donors who initially express an interest in donating a particular organ type might be entirely unaware of the possibility of donating the other organ type, or might be only partially informed on this process through their own research, alternate choice organ counselling is necessary such that those individuals who proceed to become NDA donors make fully informed choices over which organ type they donate. While this necessarily provides the potential NDA donor with more information than if they were counselled on only one kind of organ, this is unlikely to overwhelm them or impede their decision‐making, but would instead improve the autonomy of their subsequent choice. This also resembles the broad positive duty of doctors to counsel patients on all management options (such as watchful waiting, medication or surgery) for a particular condition rather than only counselling them on a single option such that those patients can make fully informed, and therefore autonomous, decisions regarding their care.^2^

### 4.2 Non‐Maleficence

Non‐maleficence requires doctors, through their medical interventions, to avoid causing intentional and unnecessary harm to patients. The process of living organ donation (directed or non‐directed, and altruistic or otherwise) by definition reduces the health of the donor as it removes from them an organ that is supporting their health by adequately functioning. Non‐maleficence, therefore, is in tension with the duty to respect the autonomous decision of potential living donors. It is generally viewed that, in potential living donors who are deemed to be psychologically and psychiatrically well, who have a sufficient degree of physical health [7, 8], and who are not being coerced or subjected to undue pressure, the duty to respect their autonomy should prevail and they should be allowed to become living donors.

The risk profile of living kidney donation and living liver lobe donation are not identical. With regard to nephrectomy (for living kidney donation), surgical mortality is roughly 1 in 3000 donors, and is higher in men than in women (5.1 vs 1.7 per 10,000 donors), while the long‐term risk of death is no higher for living donors than for age‐ and comorbidity‐matched patients [15]. The rates of postoperative complications (surgical morbidity), particularly major complications, are low [16]. While there is a generally small increase in absolute risk of end‐stage renal disease [17, 18, 19], the risk might be higher for younger donors [20] and those with a family history of end‐stage renal disease (because genetic factors—including Bangladeshi, African, and Caribbean ethnicity—increase the risk of end‐stage renal disease for both living donors and non‐donors) [21].

With regard to living liver lobe donation, due to the comparatively low total number of donations to date, the risk profile of this kind of donation is less accurately understood than that of living kidney donation. The literature suggests that overall surgical mortality for all kinds of living liver lobe donation (right lobectomy, left lobectomy, and left lateral segmentectomy) in the US and Europe is approximately 0.2%, but mortality from right lobe donation (0.23%–0.5%) is higher than that of left lobe donation (0.05%–0.21%) [8]. As this difference in mortality is due to the extent of the resection, NDA liver lobe donations usually constitute left lateral segmentectomy [22], for which the surgical mortality is lower than that of left lobectomy, and therefore exposes the NDA donor to the lowest risk. The rates of postoperative complications (surgical morbidity) of donor left lateral segmentectomy are substantially lower than those of donor right lobectomy and left lobectomy [23], but are higher than those of nephrectomy. Because the liver is the only visceral organ with the capacity to regenerate, and because residual livers are able to restore their lost mass following partial hepatectomy, the long‐term risk of advanced liver disease following living liver lobe donation is very low [24].

Accordingly, the risk of surgical mortality and morbidity is higher in living liver lobe donation than in living kidney donation, while the risk of long‐term negative health outcomes as a result of living donation is higher in living kidney donation than in living liver lobe donation (particularly for younger donors). Therefore, if potential NDA donors are conceptualised as potential NDA donors of either a kidney or a liver lobe, the duty of non‐maleficence would require doctors to favour the option that poses the lowest risk of harm to each specific donor. For example, a younger person who initially expresses an interest in becoming a NDA kidney donor would be counselled about the associated risks of both living kidney donation and living liver lobe donation, and the doctor's preference (on grounds of non‐maleficence) for the potential overall lower risk associated with living liver lobe donation would be expressed to them. In another example, an older person who initially expresses an interest in becoming a NDA liver lobe donor would be counselled about the associated risks of both living liver lobe donation and living kidney donation, and the doctor's preference (on grounds of non‐maleficence) for the potential overall lower risk associated with living kidney donation would be expressed to them. In addition, such communication of the risk profile of both organ types in NDA donation—including the fact that the risk of profile of living liver lobe donation is less well understood than that of living liver lobe donation due to the smaller total number of living liver lobe donors to date—is necessary for the patient to be fully informed when making their decision, and is therefore also necessary to respect their autonomy.

One potential threat to non‐maleficence posed by alternate choice organ counselling is that the potential donor might go on to become a serial NDA donor by donating a kidney after a liver lobe, or vice versa. Serial donation subjects the donor not only to both risk profiles of living donation of each organ type, but also to a compounded risk in the second donation due to having previously undergone major surgery in the first. If alternate choice organ counselling alerts the potential donor of the possibility of NDA donation of the other organ type, the donor might form the intention to ultimately become a serial donor. Had they not formed this intention in the absence of alternate choice organ counselling, then alternate choice organ counselling would expose them to greater total harm, which would defy the duty to non‐maleficence. While publicly available data regarding serial donation is scarce, the number of serial donors appears to be very low internationally (e.g., between 1981 and 2021 in the United States, 11 living liver lobe donors subsequently donated a kidney, and two living kidney donors subsequently donated a liver lobe [25]; and, between 2007 and 2018 in Turkey, 5 living liver lobe donors subsequently donated a kidney) [26]. Accordingly, the lack of evidence pertaining to the health outcomes of serial donation further defies the duty of non‐maleficence of a system that generates (even if unintentionally) serial donation.

### 4.3 Justice

The principle of justice requires doctors to ensure that the benefits and costs of actions are fairly distributed between patients. NDA donation of both kidneys and liver lobes directs these donor organs to patients who are in need of them, and therefore serves to promote justice.

With regard to NDA kidney donation, the UK Living Kidney Sharing Scheme (UKLKSS) [3] allows kidneys to be ‘shared’ across the entire UK. This mechanism promotes justice in at least two ways: first, it widens the pool of potential donors and recipients from that of the regional Transplant Centre which the donor initially approaches to the entire UK, which both increases the chances that the most in‐need recipient will be matched with a suitable donor, and that the donor‐recipient matches will be maximally suitable; second, the Paired/Pooled Donations (PPDs) and Altruistic Donor Chains (ADCs) made possible by the UKLKSS allow transplantations that might not take place otherwise if these sharing mechanisms were not available, thereby ‘unlocking’ additional transplantations.

With regard to NDA liver lobe donation, no equivalent sharing scheme is currently available in the UK. Accordingly, justice is not promoted to the same degree as by the UKLKSS, for two reasons: first, the pool of potential donors and recipients is restricted to that of the regional Transplant Centre which the donor initially approaches, which both reduces the chances that the most in‐need recipient in the UK will be matched with a suitable donor, and that the donor‐recipient matches will be maximally suitable (currently, there are no such Centres in Wales, Scotland or Northern Ireland. While potential NDA donors who live in these countries are able to donate in the Centres in England, this is a substantial barrier which might inhibit donations) [7]; second, the lack of PPDs and ADCs prevents the unlocking of additional transplantations that would otherwise not take place.

Accordingly, while NDA donation of both kidneys and liver lobes can respond to the regional need for such donor organs, the mechanisms of the UKLKSS promote justice in NDA kidney donation to a greater degree than that achieved by the current mechanisms in NDA liver lobe donation.^3^ This asymmetry has two effects with regard to justice when potential NDA donors are conceptualised as potential NDA donors of either a kidney or a liver lobe: first, the alternate choice organ counselling that becomes necessary would inform potential donors of this asymmetry, which might cause potential donors who intend to maximise the utility of their donation to change their decision regarding which organ to donate to achieve this (additionally, for potential donors with this kind of intention, this counselling is necessary for their choice to be informed and, therefore, for their autonomy to be respected); second, it generates the requirement for the lists of those in need of a kidney donation and those in need of a liver donation to be merged into a single standalone list and ordered according to clinical need for the specific purpose of NDA donation. This is so that potential NDA donors of either a kidney or a liver lobe can be matched with individuals in greatest need, regardless of which organ type they require, whilst the number of transplantations that can be unlocked through the UKLKSS can be maximised, such that the greatest utility can be generated from each individual NDA donation. This matching will depend on the characteristics of those individuals in need of kidney donation or liver donation, the characteristics of the potential NDA donors, and the matching suitability of these individuals, at any one time. For example, at one particular point in time, it might be the case that greater utility could be generated by a specific NDA donor donating a kidney to a highly suitable recipient and unlocking an ADC of three transplantations than by donating a liver lobe to a single recipient who is a less suitable match. Or, at another particular point in time, it might be the case that greater utility could be generated by a specific NDA donor donating a liver lobe to a highly suitable recipient who is in great clinical need than by donating a kidney to a less suitable recipient without unlocking additional transplantations through PPD or ADC.

### 4.4 Beneficence

Beneficence requires doctors to act for the benefit of the patient, such as preventing or removing harm, or the active promotion of some good, such as health. Both NDA kidney donation [27, 28, 29], and NDA liver lobe donation [30, 31], are known to often improve the psychological wellbeing of the donor, meaning both might serve to respect beneficence.

While there is currently no evidence to suggest that the improvement in psychological wellbeing is greater when one type of organ (kidney or liver lobe) is donated in a NDA manner rather than the other, specific donors might stand to benefit from greater improvements in psychological wellbeing by donating one type of organ than the other. For example, if a potential donor hopes to maximise the utility of their single NDA donation, their psychological wellbeing might be improved to a greater degree by donating a kidney and unlocking an ADC of three transplantations than by donating a liver lobe to a single recipient. Or, if a different potential donor hopes to donate an organ to a child, their psychological wellbeing might be improved to a greater degree by donating a liver lobe (since children are usually the recipients of donated left lateral segments in NDA liver lobe donation) [8] than by donating a kidney to an adult. While further research is required to assess the impact of alternate choice organ counselling on the psychological wellbeing of NDA donors, it is likely that this will improve such wellbeing and, therefore, promote beneficence in NDA donors. This is in addition to respecting the autonomy of such donors if, for example, they wish to maximise the utility of their single NDA donation, or to donate to a child.

## 5 Conclusion

Currently, recruitment of NDA donors regards these individuals as potential NDA donors of the particular organ type they initially express an interest in donating. It is possible to instead conceptualise these individuals as potential NDA donors of either a kidney or a liver lobe. Regarding potential donors in this way would require them to be thoroughly counselled on the processes of both kidney donation and liver lobe donation, such that they can subsequently choose which (if any) of these organs to donate as NDA donors. This can be referred to as ‘alternate choice organ counselling.’

Using the Principlism framework, this paper finds multiple strong ethical reasons to carry out alternate choice organ counselling for potential NDA donors of kidneys or liver lobes: the duty to respect autonomy requires alternate choice organ counselling such that the potential donor's decision to become a NDA donor of a particular organ type is fully informed; the duty of non‐maleficence requires alternate choice organ counselling such that the harm subjected to the donor through living donation can be minimised (although such counselling might generate serial NDA donors, which would expose them to greater total harm); the asymmetry in the degree to which the UKLKSS and living liver lobe donation mechanisms promote justice requires alternate choice organ counselling for potential donors who wish to maximise the utility of their single NDA donation; finally, alternate choice organ counselling is likely to promote beneficence in potential NDA donors.

## 6 Recommendations for Policy and Practice

The findings of this paper, which is restricted to an ethical analysis, suggest that, rather than regarding potential NDA donors as potential NDA donors of the particular organ type they initially express an interest in donating, it might be ethically beneficial for these individuals to instead be conceptualised as potential NDA donors of either a kidney or a liver lobe. This would require these individuals to be provided with alternate choice organ counselling during the process of assessment that forms their recruitment.

Currently, recruitment of NDA kidney donors and recruitment of NDA liver lobe donors are entirely separate processes delivered by the relevant Donor Team (the Kidney Donor Team or the Liver Donor Team, according to which organ type the potential donor initially expresses an interest in donating) [4, 7]. The findings of this ethical analysis suggest that it might be ethically beneficial for current practices to be changed such that all potential NDA donors of both kidneys and liver lobes are recruited via a single common pathway such that they can receive counselling on both types of living organ donation. Following this counselling, potential donors who wish to progress towards donation of either a kidney or a liver lobe could be recruited by the relevant Donor Team and undergo the necessary assessment. For example, in the UK, this single common pathway might be facilitated by a central NHS body such as NHS Blood & Transplant.^4^ Primary quantitative research on the predicted impact of this restructuring on health outcomes and cost‐effectiveness, and qualitative research on the perspectives of potential donors, the public generally, and the Donors Teams of both organ types on such restructuring, should be undertaken.

Another suggested change to existing practices is generated from the asymmetry in the degree to which the UKLKSS and living liver lobe donation mechanisms promote justice. This asymmetry might be ameliorated by merger of the lists of those in need of a kidney donation with the list of those in need of a liver donation into a single standalone list ordered according to clinical need for the specific purpose of NDA donation. This is so potential NDA donors of either a kidney or a liver lobe can be matched with individuals in greatest need regardless of which organ type they require, whilst the number of transplantations that can be unlocked through the UKLKSS can be maximised, to generate the greatest utility from each single NDA donation. The availability of this merged list at the beginning of NDA donor recruitment—specifically at the point of the initial single common pathway in which potential NDA donors receive counselling on both types of living organ donation—might help to inform that counselling and, therefore, the potential NDA donor's choice of which organ type to donate. However, such merger into a single standalone list poses various challenges, such as how clinical urgency can be accurately compared across the two organ types, and the logistical challenges of donor organs being offered across large geographical areas. Because no previous examples exist of such a merger from other health systems, primary research should first be undertaken to assess the clinical and logistical feasibility of this potential restructuring.

## Conflicts of Interest

Richard C. Armitage is a non‐directed altruistic kidney donor.

## Data Availability

The author has nothing to report.

## Endnotes

1 Living kidney donations can be ‘shared’ across the UK through the UK Living Kidney Sharing Scheme (UKLKSS) [3]. This contains linked donor‐recipient pairs which consist of an individual in need of a kidney (recipient) and another individual (a loved one) who is willing to donate one (donor), but the pair's tissue incompatibility prohibits a donation between them. Through the UKLKSS, incompatible linked donor‐recipient pairs are ‘matched’ with other such pairs that, in some combination, are collectively compatible for kidney sharing. In Paired‐Pooled Donations, two‐way (paired donation) sharing occurs between two linked pairs, while three‐way (pooled donation) sharing occurs between three linked pairs. Although such donations are undoubtedly virtuous, and despite the donor having no prior relationship with the recipient, they are not described as ‘altruistic’ because the donor's loved one, with whom they have formed a linked donor‐recipient pair, receives an organ in return. In Altruistic Donor Chains, individuals not in linked pairs who wish to donate a kidney without a loved one receiving one in return donate to a recipient in a linked donor‐recipient pair to trigger a chain of multiple donations. Such donations are considered to be ‘altruistic’ because the donor has no prior relationship with the recipient, and has not formed a linked donor‐recipient pair with a loved one. If tissue incompatibility prevents the formation of an Altruistic Donor Chain, individuals not in linked pairs who wish to donate a kidney without a loved one receiving one in return can donate to an unspecified individual without the formation of an Altruistic Donor Chain. While there is currently no equivalent sharing scheme for living liver lobe donations in the UK (meaning no Paired‐Pooled Donations or Altruistic Donor Chains are possible for living liver lobe donations), donors are able to donate to unspecified individuals as non‐directed altruistic donors. In these donations, donors have no prior relationship with the recipient, and the donor has not formed a linked donor‐recipient pair with a loved one.

2 Although potential NDA donors are not unwell patients who visit their doctor with the intention of improving their health, they similarly visit the Donor Team with a health‐related intention that requires the doctor's expertise. Accordingly, the broader positive duty to counsel the individual on all available options to facilitate fully informed and, therefore, autonomous choices applies to both cases.

3 The absence of an equivalent sharing scheme for living liver lobe donation is likely due to the prevalence of living liver lobe donation (31 donations between 01 April 2023 and 31 March 2024) being much lower than that of living kidney donation (907 donations between the same dates) [2].

4 Currently, both blood and plasma donation are advertised by NHS Blood and Transplant towards those who are interested in donating blood products (both online and in Donor Centres [32], such as via poster displays and opportunistically by members of staff), although the process and inherent risk of solid organ donation are substantially greater than those of donation of blood products.
